# Cerebrospinal fluid cytokines in Lyme neuroborreliosis

**DOI:** 10.1186/s12974-016-0745-x

**Published:** 2016-10-18

**Authors:** Annukka Pietikäinen, Mikael Maksimow, Tommi Kauko, Saija Hurme, Marko Salmi, Jukka Hytönen

**Affiliations:** 1 Department of Medical Microbiology and Immunology, University of Turku, Kiinamyllynkatu 13, FI-20520 Turku, Finland; 2 Turku Doctoral Programme for Molecular Medicine, Turku, Finland; 3 MediCity Research Laboratory, University of Turku, Turku, Finland; 4 Department of Biostatistics, University of Turku, Turku, Finland; 5 Department of Clinical Microbiology and Immunology, Turku University Hospital, Turku, Finland

**Keywords:** Borrelia, Lyme disease, Neuroborreliosis, Cytokine profiling, CXCL13

## Abstract

**Background:**

Lyme neuroborreliosis (LNB) is one of the manifestations of Lyme disease. Although it is known that immune reaction of LNB patients is dominated by Th1 and Th2 responses and patients have elevated numbers of B cells in their cerebrospinal fluid (CSF), not all the cells involved in inflammation and cytokine secretion have been characterized. The current diagnostics of LNB is based on intrathecal production of antibodies. In recent years, the measurement of chemokine CXCL13 concentration from the CSF has been introduced as a new promising diagnostic tool for LNB to complement the antibody-based diagnostic methods. A few other cytokines have also been analyzed as possible diagnostic markers. However, multiplex analyses simultaneously evaluating the concentrations of a large number of different cytokines in the CSF of LNB patients have been lacking thus far. Extensive cytokine profiling CSF samples of LNB patients would also help in understanding the complex immunopathogenesis of LNB.

**Methods:**

CSF samples were analyzed from 43 LNB patients, 19 controls, 18 tick-borne encephalitis patients, and 31 multiple sclerosis patients. In addition, CSF samples from 23 LNB patients obtained after the antibiotic treatment were examined. Altogether, the concentrations of 49 different cytokines were determined from all of the samples. The concentrations of 48 different cytokines were analyzed by magnetic bead suspension array using the Bio-Plex Pro Human Cytokine 21- and 27-plex panels, and the concentration of CXCL13 was analyzed by an ELISA based method.

**Results:**

Distinct cytokine profiles which were able to distinguish LNB patients from controls, tick-borne encephalitis patients, multiple sclerosis patients, and LNB patients treated with antibiotics were identified. LNB patients had elevated concentrations of all major T helper cell type cytokines (Th1, Th2, Th9, Th17, and Treg) in their CSF.

**Conclusions:**

Despite the great differences in the CSF cytokine profiles of different patient groups, CXCL13 still remained as the best marker for LNB. However, IL-1ra might also be helpful as a marker for the antibiotic treatment response. Concerning the immunopathogenesis, this is the first report suggesting the involvement of Th9 cells in the immune response of LNB.

**Electronic supplementary material:**

The online version of this article (doi:10.1186/s12974-016-0745-x) contains supplementary material, which is available to authorized users.

## Background

Lyme disease is the most common vector-borne disease in the Northern hemisphere. It is caused by *Borrelia burgdorferi* sensu lato spirochetes which are transmitted to humans through the bite of an infected tick vector. *B. burgdorferi* sensu lato group (later referred to as “borrelia”) comprises of several different genospecies [1] of which *B. burgdorferi* sensu stricto, *B. afzelii*, and *B. garinii* are the most common disease-causing agents. Different genopecies of borrelia are usually associated with different disease outcomes; for example, *B. garinii* is considered to be the most common cause of Lyme neuroborreliosis (LNB) in Europe [2]. The symptoms of LNB appear within a few weeks after the bite of an infected tick although, in rare cases, the development of symptoms may take a few months or even years. LNB patients may suffer, e.g., from lymphocytic meningitis, radiculoneuritis, and cranial neuritis [3, 4].

Immunopathology of LNB is not entirely understood. Patients are defined as having elevated white blood cell counts in their CSF with over 90 % of these cells being lymphocytes [4, 5]. By analyzing cytokine production in the cerebrospinal fluid (CSF) of LNB patients, it has been demonstrated that immune reactions of the patients are characterized by a Th1 type response early in the course of infection, and later during the disease, the immune defense is accompanied by a Th2 response [6]. In addition to a T cell response, CSF of LNB patients contains more B cells than the CSF of patients with other central nervous system diseases [5, 7, 8]. However, the role of other cell types in the immunopathology of LNB is less evident and not all the cells involved in inflammation and cytokine secretion have been characterized. An extensive cytokine profiling of CSF samples of LNB patients would lead to a deeper understanding of the immunopathogenesis of LNB.

The diagnosis of LNB is based on the assessment of neurological symptoms, B cell pleocytosis in the CSF, and most importantly, intrathecal production of antibodies against borrelia. Antibody-based diagnosis, however, has limitations because antibodies are absent during the early phase of the disease. Borrelia-specific antibodies may also persist long after a patient has been treated which complicates the discrimination of an acute reinfection from a previously treated and cured disease [4].

Recently, measurement of the concentration of a chemokine CXCL13 in the CSF samples of suspected LNB patients has been introduced as a new diagnostic tool for the infection. CXCL13 levels of LNB patients are highly elevated when compared with healthy controls or patients suffering from other neurological conditions [9–17]. Also, the levels of a few other cytokines have been studied in the CSF of LNB patients [6, 8, 16, 18–24], but importantly, a multiplex analysis of CSF samples of LNB patients including a large panel of different cytokines has been lacking thus far.

In this study, we compared the concentrations of 49 different cytokines among CSF samples of LNB patients, non-LNB controls, and patients suffering from other neurological conditions. Our main aims were to gain new information on the immunopathology of LNB and to identify new biomarkers for the laboratory diagnostics of the infection. We also measured the CSF cytokine concentrations after antibiotic treatment of LNB to evaluate the applicability of the molecules as markers of treatment response. As a result of the profiling, we identified distinct cytokine profiles in the CSF of LNB patients which suggest the involvement of B cells and all major T helper cell types in the immunopathology of LNB. Chemokine CXCL13, however, still remains as the best biomarker for LNB.

## Methods

### Patient samples

CSF samples used in this study were from patients identified retrospectively from the laboratory information-management system of our laboratory. The samples were from 43 LNB patients (samples drawn before the onset of antibiotic treatment), 19 non-LNB control subjects, 18 tick-borne encephalitis (TBE) patients, and 31 multiple sclerosis (MS) patients. LNB and TBE patients were diagnosed as previously described [9]. The non-LNB controls were samples that were sent to our laboratory for borrelia antibody analysis and that were found to be borrelia antibody negative. MS patients were clinically diagnosed cases from the local university hospital. In addition, follow-up samples obtained from 23 of the 43 LNB patients after the antibiotic treatment (6–380 days) were analyzed. LNB patients were treated with intravenous ceftriaxone and/or oral doxycycline.

CSF samples were obtained from the patients by lumbar puncture, sent to our laboratory at room temperature, and stored at 4 °C until diagnostic assays were performed. After the initial analyses, the samples were stored in −20 °C until further usage.

### Multiplex cytokine analysis

Cytokine levels in all CSF samples were determined by magnetic bead suspension array using the Bio-Plex Pro Human Cytokine 21- and 27-plex panels (Bio-Rad Laboratories, Hercules, CA, USA) as previously described [25–27]. The panels included the cytokines listed in Table 1.

**Table 1:** Cytokines included in the study

IL-1α	IL-16	TNF-β
IL-1β	IL-17	HGF
IL-1ra	IL-18	LIF
IL-2	CXCL1/Groα	M-CSF
IL-2ra	CXCL9/MIG	MIF
IL-3	CXCL10/IP-10	β-NGF
IL-4	CXCL12α/SDF-1α	SCF
IL-5	CCL2/MCP-1/MCAF	SCGF-β
IL-6	CCL3/MIP-1α	TRAIL
IL-7	CCL4/MIP-1β	PDGF-bb
IL-8/CXCL8	CCL5/RANTES	FGF basic
IL-9	CCL7/MCP-3	G-CSF
IL-10	CCL11/Eotaxin-1	GM-CSF
IL-12(p40)	CCL27/CTACK	VEGF
IL-12(p70)	IFN-α2	
Il-13	IFN-γ	
IL-15	TNF-α	CXCL13/BCA-1^a^

The cytokine concentrations were analyzed with magnetic bead suspension array using the Bio-Plex Pro Human Cytokine 21- and 27-plex panels

^a^The concentrations of chemokine CXCL13 were analyzed with an ELISA-based method

Recombinant cytokines included in the kit were used to generate standard curves for each analyte. Individual concentration readings which remained below the measuring range of the assay were given a value that corresponded to a half of the lowest standard concentration of the respective cytokine analysis, and on the other hand, concentrations over the detection range were given a value that was 1.5 times the highest concentration of the standard.

### CXCL13 measurement

CXCL13 levels in the CSF samples were measured as previously described [9] with a human CXCL13 kit (Quantikine; R&D Systems, Minneapolis, MN, USA) according to the manufacturer’s instructions. Samples with a concentration below the standard curve of the assay were given a value that corresponded to a half of the value of the lowest point of the standard.

### Statistical analyses

Continuous variables were characterized using medians and range of values, and in case of categorical variables, frequencies and percents were used. Mann-Whitney *U* test was used to test the difference in age between the groups, and for sex, Pearson’s chi-squared test was used.

Differences in cytokine concentrations between patient groups (LNB, non-LNB controls, TBE, and MS) were tested using independent samples *t* test for logarithmic transformed variables, and Bonferroni’s method was used to correct the *p* values for multiplicity. The results are expressed using *p* values and mean difference between groups with 95 % confidence intervals (95 % CI). To find the most important cytokines to distinguish the patient groups the random forests method was used [28]. The cytokines were ordered using variable importance, which is calculated by the mean decrease in the Gini coefficient. Gini coefficient represents the contribution of each cytokine to the homogeneity of the results of random forests. The higher the variable importance is the better the cytokine is able to discriminate the groups. The distributions of different patient groups of nine cytokines with the best variable importance were presented using box-plots.

Paired samples *t* test was used to test the difference in cytokine concentrations before and after treatment in LNB patients. Logarithmic transformation was made for the data. The results are expressed using *p* values and mean of difference between the measurements (before-after) with 95 % confidence intervals (95 % CI). Nine cytokines with the smallest *p* values were presented using box-plots. The control group was added to these box-plot figures as well to illustrate the difference with controls although this group was not included in the analyses.

In all of the analyses of cytokines, the natural logarithmic transformation was used to achieve the normality of the distributions and all of the results and figures are presented using transformed variables. *p* values less than 0.05 were considered as statistically significant. Statistical analyses were carried out using SAS System for Windows, version 9.4 (SAS Institute Inc., Cary, NC; USA), and all figures were drawn with R 3.0.2 or 3.2.4 (R Foundation for Statistical Computing, Vienna, Austria).

## Results

### Demographic features of patients

The demographic features of the patients are presented in Table 2. The age of LNB patients varied from two to 82, with the median age being 54. Median ages of the non-LNB control patients, TBE patients, and MS patients were 56 (range 17–78), 62.5 (range 18–80), and 33 (range 21–72), respectively. Male patients were dominant in the LNB (65 %, *n* = 28), TBE (67 %, *n* = 12) and non-LNB control patient (63 %, *n* = 12) groups while there were more female (77 %, *n* = 24) than male patients in the MS group. Nevertheless, there was no statistically significant difference in the distribution of age (*p* = 0.817) or the sex ratio (*p* = 0.741) between the groups.

**Table 2:** Demographic features of the patients

	LNB	Non-LNB controls	TBE	MS
Median age	54	56	62.5	33
Age range	2–82	17–78	18–80	21–72
Male	*n* = 28 (65 %)	*n* = 12 (63 %)	*n* = 12 (67 %)	*n* = 7 (23 %)
Female	*n* = 15 (35 %)	*n* = 7 (37 %)	*n* = 6 (33 %)	*n* = 24 (77 %)

### Specific cytokine profiles distinguish the patient groups from each other

The main goal of this study was to characterize the expression profile of a large panel of different cytokines in the CSF of LNB patients and to compare the observed profile to the cytokine levels of non-LNB controls and patients suffering from other neurological conditions.

**Fig. 1:**
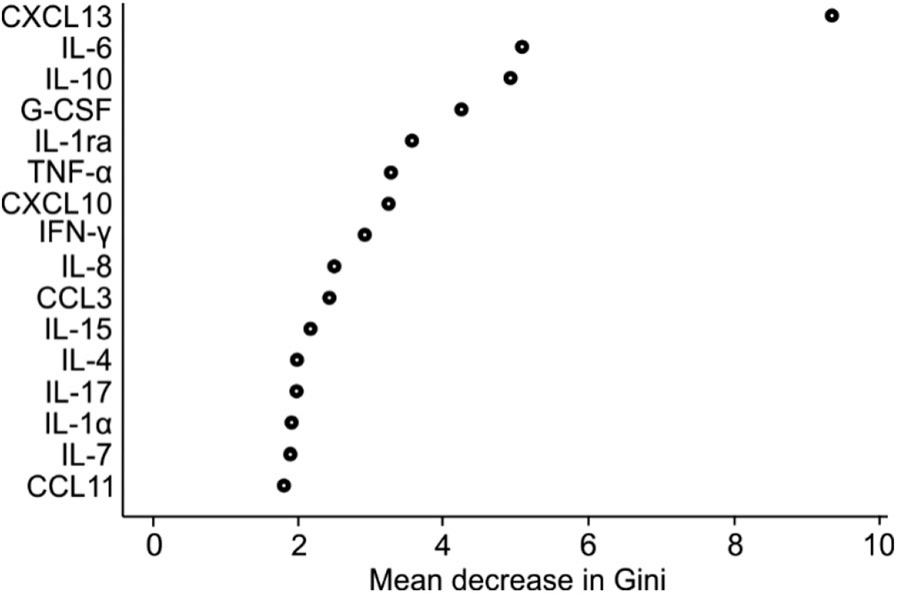
The cytokines with the highest variable importance when distinguishing patient groups from each other. Variable importance is given by the mean decrease in the Gini coefficient, which represents the contribution of each cytokine to the homogeneity of the results of the random forests. The concentrations of all studied cytokines in the CSF of patient groups (LNB, TBE, MS, and non-LNB controls) were compared. Sixteen best cytokines to distinguish all groups from each other are presented in the figure in descending order. The higher the importance is, the better the respective cytokine is able to discriminate the groups

Cytokines IL-2 and IL-5 and chemokine CCL7/MCP-3 did not reach the detection limit of the multiplex assay in the most of the measured samples, and thus, they were excluded from further analyses. Nevertheless, several other cytokines proved to be interesting in terms of separating the LNB patients from the other study groups. According to the variable importance (mean decrease in the Gini coefficient) calculated with the random forests -method, the best nine markers to distinguish all patient groups from each other were CXCL13, IL-6, IL-10, G-CSF, IL-1ra, TNF-α, CXCL10, IFN-γ, and IL-8 (Fig. 1).

**Fig. 2:**
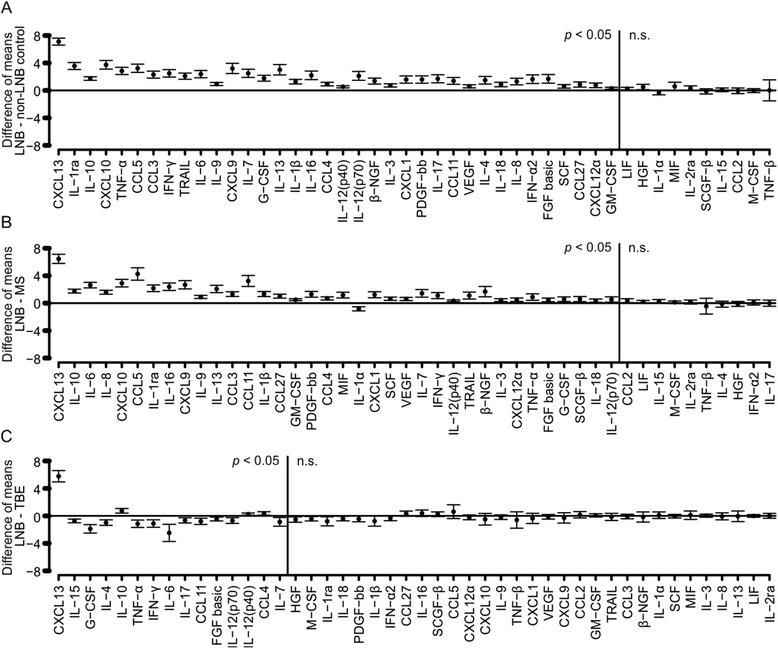
Pairwise comparisons of cytokine concentrations (in logarithmic scale) in the CSF of LNB patients and other patient groups. Pairwise comparisons between LNB patients and non-LNB controls, MS patients, and TBE patients were made using independent samples *t* test and Bonferroni’s method was used to adjust the *p* values. The cytokines were placed in order according to calculated *p* values. Difference of means between the patient groups with 95 % confidence intervals are presented for all of the cytokines. The *vertical lines* in each panel separate statistically significant differences (*p* < 0.05) and not significant differences (n.s.). **a** Comparison of LNB patients and non-LNB controls. **b** Comparison of LNB patients and MS patients. **c** Comparison of LNB patients and TBE patients

Pairwise comparisons between the groups were made to more closely analyze the differences in cytokine profiles of the groups. Specific cytokine profiles which were able to discriminate the groups from each other were detected, and the differences found were statistically significant (Fig. 2, Additional file 1). Out of the 46 analyzed cytokines, only ten (LIF, HGF, IL-1α, MIF, IL-2ra, SCGF-b, IL-15, CCL2, M-CSF, TNF-β) were unable to distinguish LNB patients from the non-LNB controls (Fig. 2a). Excluding these ten cytokines, all other studied cytokines were significantly elevated in the CSF of LNB patients when compared with the non-LNB controls. Moreover, there were only ten cytokines (CCL2, LIF, IL-15, M-CSF, IL-2ra, TNF-β, IL-4, HGF, IFN-α2, IL-17) which were unable to separate LNB patients from MS patients (Fig. 2b). LNB and TBE patients, on the other hand, showed more similarities in their cytokine profile. Yet, the levels of 15 cytokines were significantly different between these two groups (Fig. 2c). The majority of these 15 cytokines were more elevated in the CSF of TBE patients when compared with LNB patients. Further, TBE patients and MS patients were found to have particular CSF cytokine profiles which were able to separate these groups from each other and from the non-LNB controls (Additional file 1).

### Cytokine profile as an indicator of immunopathogenesis of the different conditions

Overall, TBE patients seemed to have the strongest inflammatory responses of all the groups analyzed. They had elevated concentrations of all major T helper cell type cytokines (Th1—e.g., TNF-α, IFN-γ/Th2—e.g., IL-4, IL-6/Th9—e.g., IL-9/Th17—e.g., IL-17/Treg—e.g., IL-10) in the CSF which indicates the involvement of many cell types in the immune response. LNB patients also had strong inflammatory responses and elevation of such cytokines which suggest the activation of all the above mentioned T cell types. There were two cytokines (IL-15 and LIF) which were elevated only in the CSF of TBE patients but not in the CSF of LNB patients when compared with non-LNB control patients (Additional file 2). On the contrary, no cytokines which would have been elevated in the CSF of LNB patients but not in the CSF of TBE patients when compared with non-LNB controls could be detected.

The concentrations of cytokines in the CSF of MS patients were not nearly as high as in the CSF of LNB and TBE patients. However, also the cytokine profile of MS patients suggests the involvement of Th1, Th2, and Th17 cells in the inflammatory response. Chemokine CCL2 and cytokine IL-1α were the only markers the concentrations of which were significantly different in the CSF of MS patients but not in the CSF of LNB or TBE patients when compared with the non-LNB controls (Additional file 2).

### CXCL13 is a superior biomarker for LNB

**Fig. 3:**
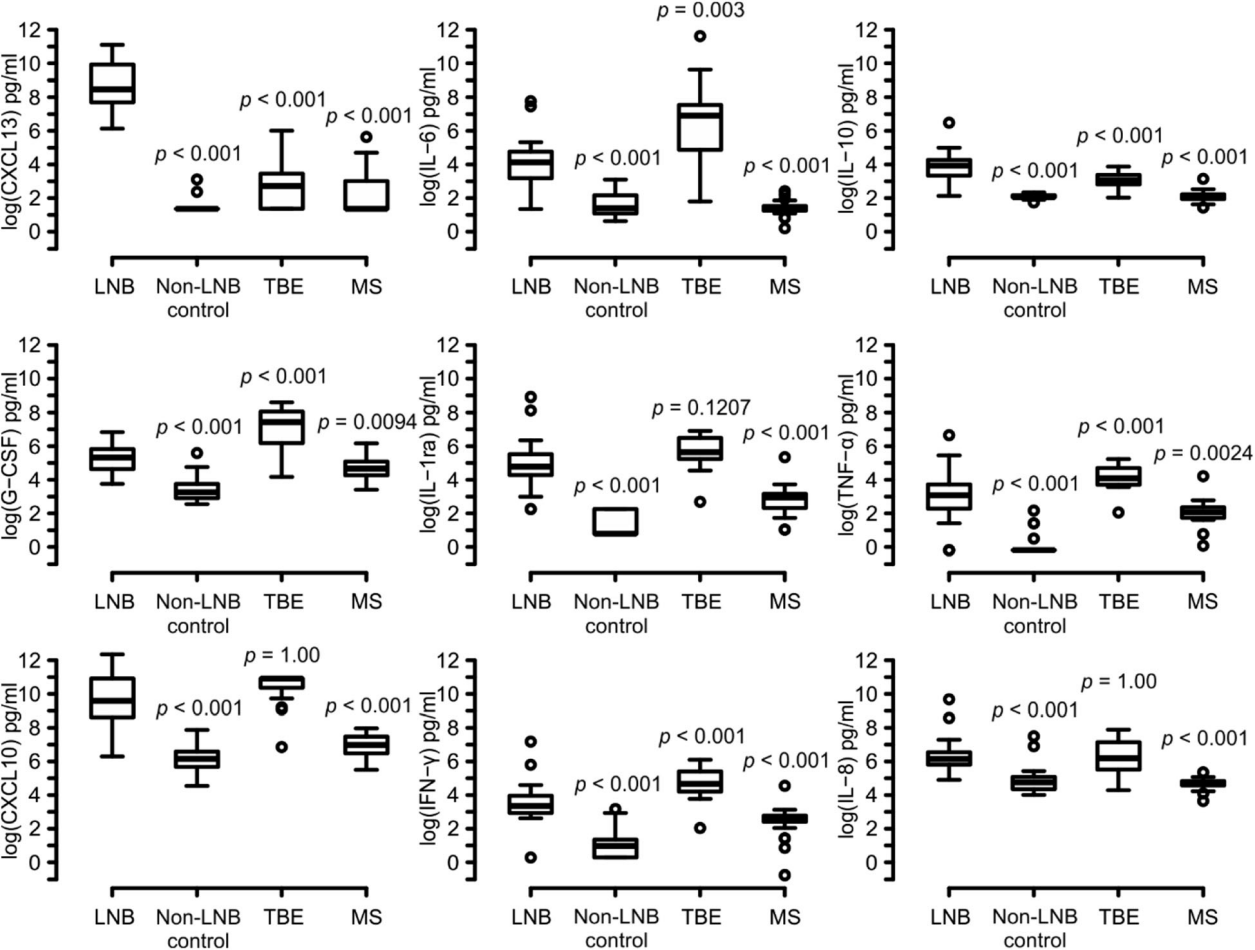
Nine most powerful cytokines to distinguish all patient groups from each other. According to variable importances (mean decrease in the Gini coefficient), nine most powerful cytokines to separate the patient groups from each other are presented more in depth. Cytokine concentrations in the CSF are presented as picogram per milliliter on a logarithmic scale. *p* values represent statistical difference of the groups compared to LNB patients (independent samples *t* test with Boferroni’s correction)

Nine of the best cytokines to distinguish all patient groups from each other according to the variable importance (CXCL13, IL-6, IL-10, G-CSF, IL-1ra, TNF-α, CXCL10, IFN-γ, and IL-8) are presented more in depth (Fig. 3). Many statistically significant differences in the levels of each of cytokine were found between the patient groups (Fig. 3, Additional file 2). However, the majority of the cytokines were the most elevated in the CSF of the TBE patients. Indeed, out of the above mentioned nine cytokines, cytokine IL-10 and chemokine CXCL13 were the only markers with a higher median concentration in the CSF on LNB patients when compared with TBE patients. Importantly, CXCL13 was the only cytokine whose concentration showed no overlap between LNB (lowest value 460 pg/ml) and all other groups (highest value 406.4 pg/ml) (Additional file 2).

**Fig. 4:**
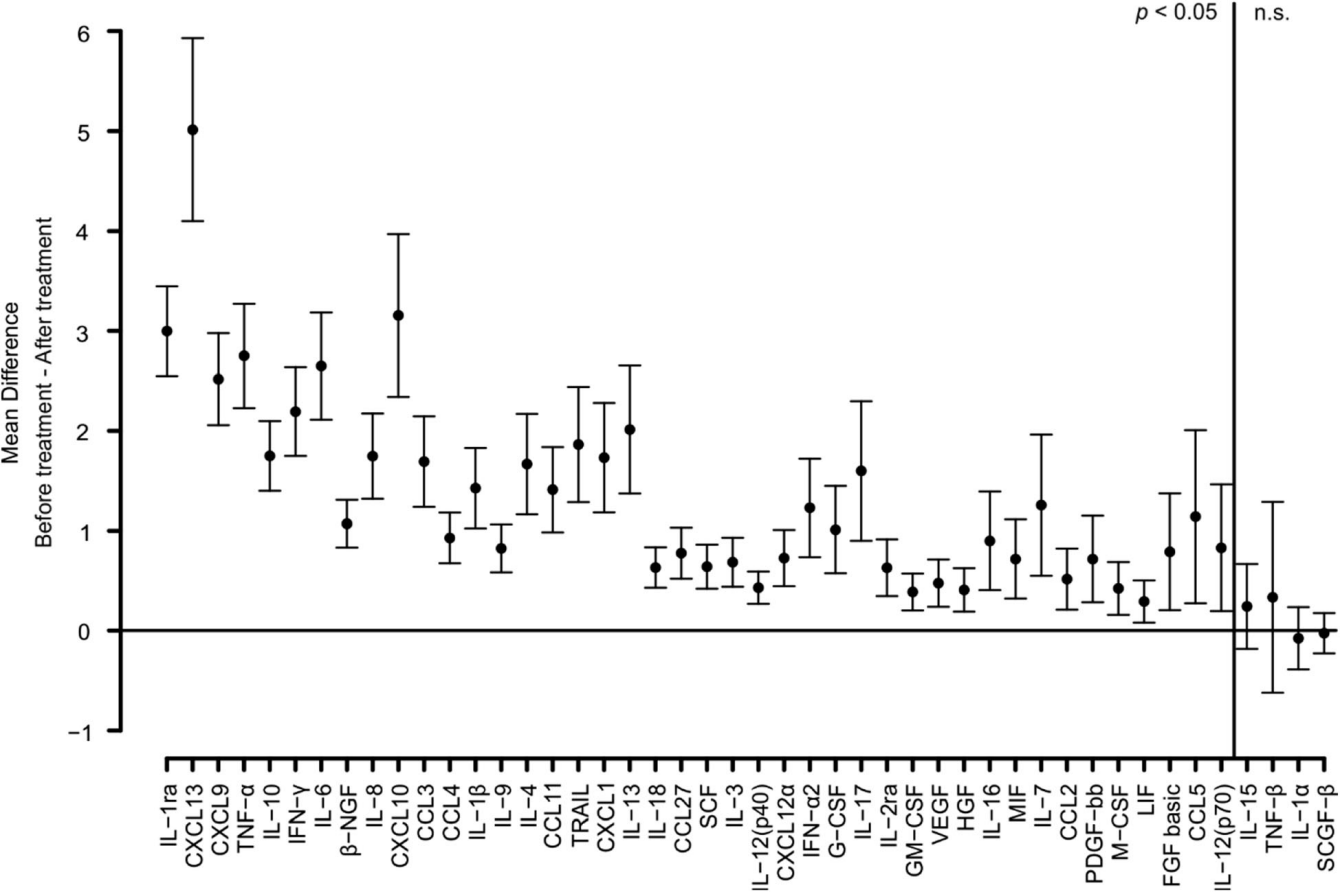
Pairwise comparison (in logarithmic scale) of cytokine concentrations between LNB patients before and after antibiotic treatment. Differences in cytokine concentrations before and after treatment in LNB patients were analyzed using paired samples *t* test. The cytokines were placed in order according to calculated *p* values. The mean of difference between the measurements before and after treatment with 95 % confidence interval is presented. The *vertical line* separates statistically significant differences (*p* < 0.05) and not significant (n.s.) differences

**Fig. 5:**
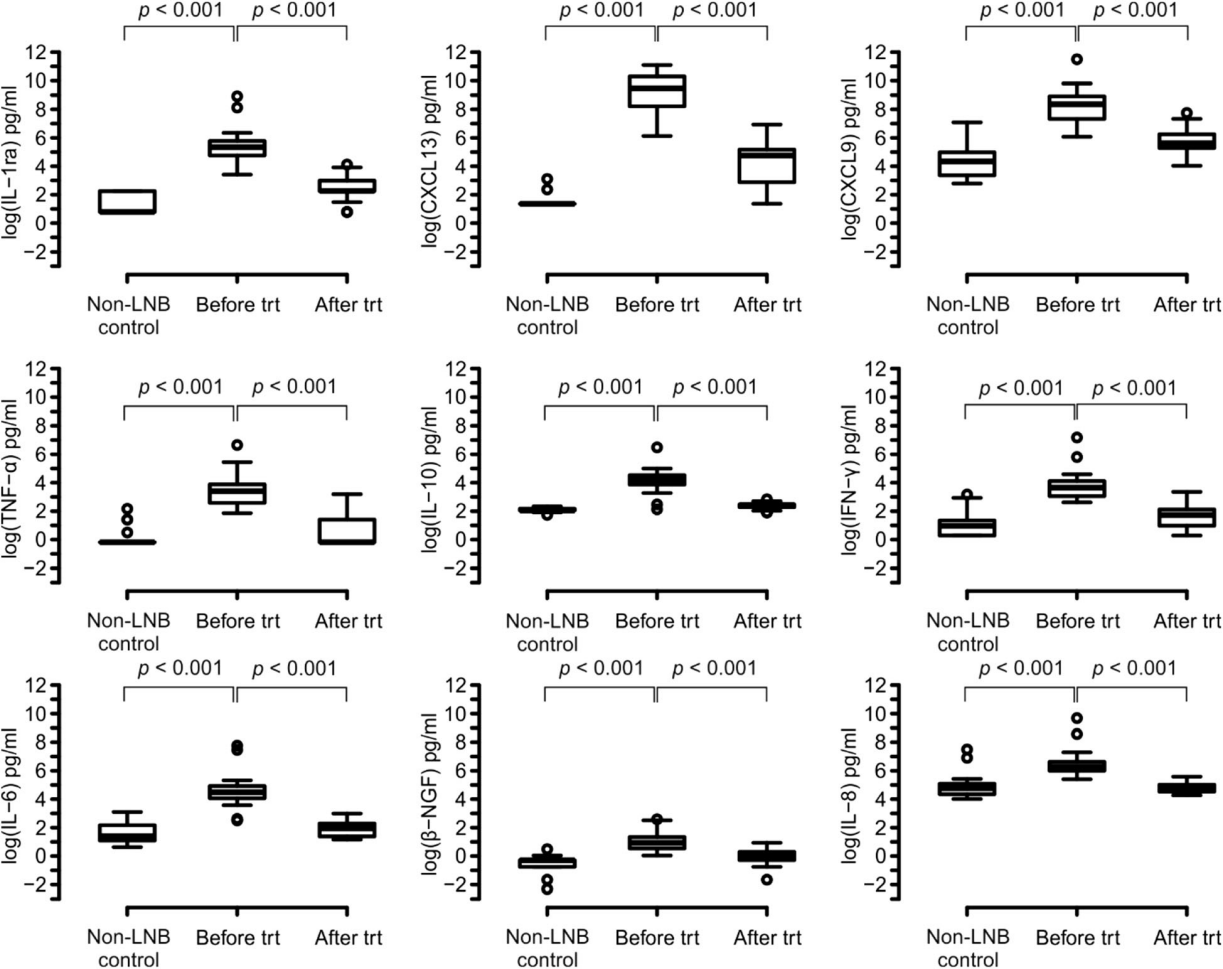
Nine most powerful cytokines as markers of antibiotic treatment response. According to analysis made in Fig. 4, nine best cytokines to differentiate CSF samples of LNB patients after the antibiotic treatment from samples taken before the treatment are presented more in depth. Cytokine concentrations in the CSF are presented as picogram per milliliter on a logarithmic scale

In order to identify biomarkers of LNB treatment response, we also evaluated the effect of antibiotic therapy on the concentrations of different cytokines. Forty-two of the cytokines analyzed were significantly different in LNB patients after the antibiotic treatment when compared with the situation before starting the treatment (Fig. 4). As expected, all of these 42 cytokines were elevated before the treatment and the levels declined statistically significantly after the course of antibiotics. According to this initial analysis, nine best markers are presented more in depth in Fig. 5. CXCL13 and IL-1ra were, based on the statistical analysis, the most useful molecules as biomarkers of LNB treatment response (Fig. 4, Fig. 5).

## Discussion

The laboratory diagnostics of LNB is based on the CSF lymphocytic pleocytosis and the detection of intrathecal borrelia-specific antibody production. Although, in the majority of cases, this approach is highly reliable, it has certain drawbacks. First of all, antibodies are absent in the early phase of the disease, and secondly, they may persist in the CSF of LNB patients long after the antibiotic treatment [4]. Because of these limitations in the antibody-based diagnostics of LNB, novel biomarkers for the disease are required.

In recent years, chemokine CXCL13 has been introduced as a new biomarker for LNB. The levels of CXCL13 in the CSF of LNB patients are highly elevated when compared with healthy controls or patients suffering from other neurological conditions [9–17]. Other conditions where elevated CXCL13 levels can be detected include, for example, neurosyphilis and CNS lymphoma [9, 10, 16, 29–32]. In addition to CXCL13, the concentrations of around 30 other cytokines in the CSF of LNB patients have previously been analyzed and studied as biomarkers for LNB [6, 8, 18–22]. To complement the thus far described cytokine profiles, we performed a multiplex analysis measuring the levels of 48 different cell signalling molecules in the CSF of LNB patients, non-LNB control patients, TBE patients, and MS patients. The levels of 36 cytokines were significantly higher in the CSF of LNB patients when compared with the non-LNB control patient group. In addition, 15 cytokines were able to discriminate LNB patients from TBE patients and the levels of 36 cytokines were significantly different in LNB patients when compared with MS patients. However, in spite of the statistical differences in many of the cytokines between LNB patients and the other patient groups, CXCL13 was the only molecule which explicitly discriminated LNB patients from all other study groups. This result is in accordance with previous reports [9–16] and highlights the unique role of CXCL13 as an LNB biomarker.

One of the aims of this study was to evaluate the applicability of different cytokines in the CSF as indicators of antibiotic treatment response of LNB patients. Previously, it has been shown that the levels of six different cytokines (IL-1β, IL-8/CXCL8, CXCL12, CXCL13, CCL3, and CCL4) decline after the antibiotic treatment [9, 13, 15, 21, 22]. In this study, we demonstrated that the levels of not only these 6 cytokines but also of 36 other cytokines were significantly elevated in the CSF of LNB patients when compared with the same patients after the antibiotic treatment. In addition to CXCL13, the concentration of which rapidly declines after treatment, for example, IL-1ra appeared as a potential indicator of antibiotic treatment response.

At least 21 of the previously studied cytokines were also included in our multiplex panel. The levels of IL-1/IL-1β, IL-4, IL-6, IL-7, IL-8/CXCL8, IL-10, IL-12, IL-17, IL-18, CXCL10, CXCL12, CCL3, CCL4, IFN-γ, TNF-α, and GM-CSF have been shown to be elevated in the CSF of LNB patients and our study confirms these results [6, 8, 16, 19–24]. IL-15, on the other hand, was one of the cytokines which was not elevated in the CSF of LNB patients. This result is also in line with previous studies showing no difference between the IL-15 levels of LNB patients and the control groups [23, 24]. Because most of the results achieved in this study are supported by previous publications, the technical validation of the used multiplex analysis procedure is well supported. One previous report demonstrates the elevation of CCL2 in the CSF of children suffering from LNB when compared with the controls [23]. However, CCL2 was not elevated in the CSF of our LNB study patients whose age varied between 2 and 82. Cytokine IL-13, on the other hand, was elevated in the CSF of LNB patients in this study, although a previous report has demonstrated that this cytokine is not elevated in LNB children [23]. Thus, the age of the study subjects appears to have an impact on the CSF cytokine concentrations. However, no significant age or sex related differences in cytokine concentrations were detected in this study. Two cytokines, IL-5 and IL-2, which have previously been suggested to be either elevated or downregulated in the CSF of LNB patients [8, 19, 23, 24] did not reach the detection limit in the most of our study samples. The time used for the transportation of the CSF samples to laboratories may vary considerably and, thus, lead to the observed variation in cytokine concentrations between different studies. In addition, variation between studies could be explained by differences in the handling and the storage of the CSF samples. However, overall our results demonstrate that, in addition to the previously studied cytokines, there are at least 15 more cytokines the levels of which are significantly different between the CSF of LNB patients and the CSF of non-LNB control patients.

One possible explanation for the differences found in the CSF cytokine profiles between various studies might be the distinct abilities of borrelia genospecies and strains to cause inflammation. For example, *B. burgdorferi* strains from Europe and the USA have been shown to cause different inflammation patterns. When peripheral blood mononuclear cells were stimulated with various *B. burgdorferi* strains, the US strains triggered stronger immune response than the strains from Europe. US strains induced higher levels of innate immune mediators as well as Th1-associated cytokines. The strains from Europe, on the other hand, induced stronger expression of Th17 cytokines when compared with the US strains [33]. Thus, the cytokine profile of an individual patient most probably depends, in addition to the immune system of the infected individual, on which borrelia genospecies and strain caused the disease.

Concerning the immunopathogenesis of LNB, it has been demonstrated that the CSF of LNB patients is characterized by a lymphocytic pleocytosis and, notably, LNB patients have more B cells in their CSF when compared with the CSF of patients suffering from many other neurological conditions [5, 7, 8]. Furthermore, chemokine CXCL13, which was demonstrated to be superior as a biomarker for LNB when compared with any other cytokine analyzed, is a B cell chemoattractant [34]. This suggests that B cells have an important role in the immunopathology of LNB. In addition, the CSF of LNB patients has been characterized by cytokines reflecting a Th1 type immune response early in the infection which is later accompanied by a Th2 type response [6]. Also, the involvement of Th17 and Treg immune responses in the immunopathology of LNB has been suggested [6, 23, 24, 35]. In this study, we confirm that Th1, Th2, Th17, and Treg immune responses are all involved in the immunopathology of LNB as the signature cytokines of these responses were elevated in the CSF of LNB patients when compared with the non-LNB controls. Furthermore, this study is the first report demonstrating IL-9 secretion in the CSF of LNB patients which suggests that also the quite recently described Th9 cells [36] are involved in the immunopathology of LNB. This finding is supported by an in vitro study which has previously demonstrated that the signature cytokine of Th9 response, cytokine IL-9, can be produced by borrelia stimulated macrophages [37].

A main symptom of LNB is painful meningoradiculitis [4]. The cause of the pain has remained somewhat unravelled. Cytokines IL-6, IL-8, and CCL2 have been associated with the sensation of pain and proposed to be the triggers of pain sensation in LNB [38, 39]. Also in our study, LNB patients had elevated concentrations of IL-6 and IL-8 in their CSF when compared with the non-LNB controls. However, also TBE patients had higher concentrations of IL-6 and IL-8 in their CSF when compared with non-LNB controls even though TBE is not generally characterized by meningoradiculitis [40]. The concentration of IL-6 was even higher in TBE patients when compared with LNB patients. This indicates that there have to be some other mediators as well which cause the pain sensation specifically in LNB patients but not in TBE patients. Chemokine CXCL13 has recently been linked to neuropathic pain in a mouse model [41]. The concentration of CXCL13 in the CSF of LNB patients is significantly higher when compared with non-LNB controls or TBE patients. Thus, CXCL13 might be one of the reasons behind the sensation of pain of LNB patients. In the future, it would be interesting to more closely analyze the role of CXCL13 as the causative agent of the pain sensation in LNB patients. In fact, we are conducting a clinical study where LNB patients evaluate their subjective sensation of pain on a specific scale before and after antibiotic treatment. The concentration of CXCL13 in the CSF of these patients will also be measured prior and after the treatment to analyze the correlation between CSF CXCL13 concentration and the sensation of pain.

## Conclusions

In this study, we demonstrated that there were significant differences in the CSF cytokine profiles of LNB, non-LNB, TBE, and MS patients. Yet, chemokine CXCL13 clearly differentiated LNB patients from other study groups the best with the highest variable importance and the biggest difference of means when compared with the other patient groups. This result highlights the uniqueness of CXCL13 as a biomarker of LNB. In addition, we demonstrated that, in addition to B cells, all major T helper cell types are involved in the immunopathogenesis of LNB.

## Additional files

Additional file 1: Pairwise comparisons of cytokine concentrations (in logarithmic scale) in the CSF of TBE, MS, and non-LNB patients. Pairwise comparisons were made between TBE, MS, and non-LNB patient groups using independent samples *t* test and Bonferroni’s method was used to adjust the *p* values. The cytokines were placed in order according to calculated *p* values. Difference of means between the patient groups with 95 % confidence intervals are presented for all of the cytokines The vertical lines in each panel separate statistically significant differences (*p* < 0.05) and not significant (n.s.) differences. (A) Comparison of MS patients and TBE patients. (B) Comparison of non-LNB controls and MS patients. (C) Comparison of non-LNB controls and TBE patients. (DOCX 326 kb)
Additional file 2: CSF cytokine concentrations in LNB, non-LNB, TBE, and MS patients. The median concentrations and ranges of each measured cytokine in the CSF of different patient groups are presented. Also the *p* values of statistical analyses (independent samples *t* test with Boferroni’s correction) of each studied cytokine are shown. (DOCX 22 kb)

## Abbreviations

CCL: Chemokine C-C motif ligand
CSF: Cerebrospinal fluid
CXCL: Chemokine C-X-C motif ligand
FGF basic: Basic fibroblast growth factor
G-CSF: Granulocyte colony-stimulating factor
GM-CSF: Granulocyte-macrophage colony-stimulating factor
HGF: Hepatocyte growth factor
IFN: Interferon
IL: Interleukin
LIF: Leukemia inhibitory factor
LNB: Lyme neuroborreliosis
M-CSF: Macrophage colony-stimulating factor
MIF: Macrophage migration inhibitory factor
MS: Multiple sclerosis
PDGF-bb: Platelet-derived growth factor subunit B homodimer
ra: Receptor antagonist
SCF: Stem cell factor
SCGF-β: Stem cell growth factor β
TBE: Tick-borne encephalitis
TNF: Tumor necrosis factor
TRAIL: TNF-related apoptosis-inducing ligand
VEGF: Vascular endothelial growth factor
β-NGF: β nerve growth factor
