# Supplementary figures and images for: Cerebrospinal fluid cytokines in Lyme neuroborreliosis
Source: J Neuroinflammation. 2016 Oct 18;13:273. doi: 10.1186/s12974-016-0745-x (PMC5070144; doi:10.1186/s12974-016-0745-x)

Additional file 1.


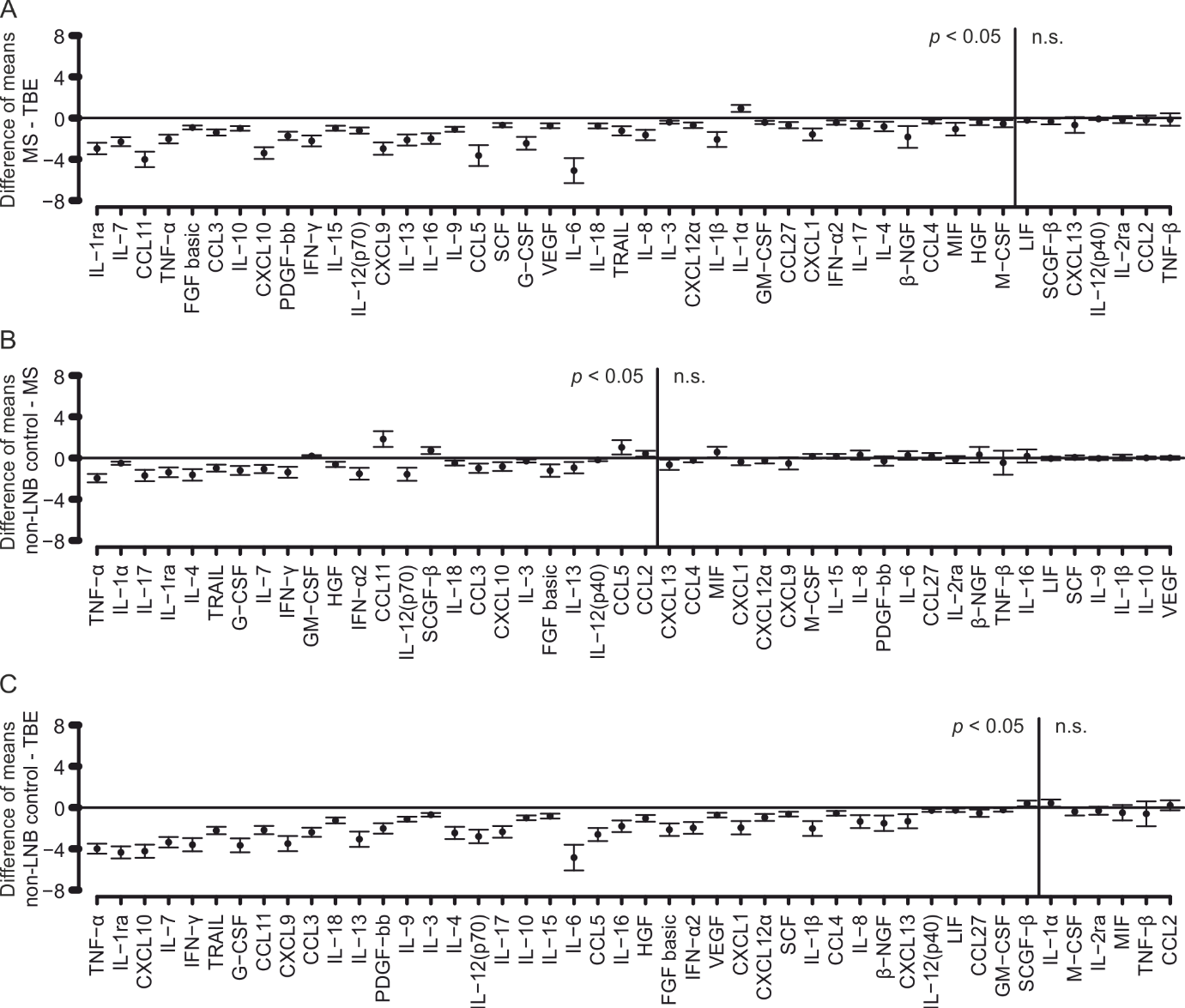

Supplement: Additional file 1: — Pairwise comparisons of cytokine concentrations (in logarithmic scale) in the CSF of TBE, MS, and non-LNB patients. Pairwise comparisons were made between TBE, MS, and non-LNB patient groups using independent samples t test and Bonferroni’s method was used to adjust the p values. The cytokines were placed in order according to calculated p values. Difference of means between the patient groups with 95 % confidence intervals are presented for all of the cytokines The vertical lines in each panel separate statistically significant differences (p < 0.05) and not significant (n.s.) differences. (A) Comparison of MS patients and TBE patients. (B) Comparison of non-LNB controls and MS patients. (C) Comparison of non-LNB controls and TBE patients. (DOCX 326 kb) [file 12974_2016_745_MOESM1_ESM.docx]
