# Supplementary material for: Cerebrospinal fluid cytokines in Lyme neuroborreliosis
Source: J Neuroinflammation. 2016 Oct 18;13:273. doi: 10.1186/s12974-016-0745-x (PMC5070144; doi:10.1186/s12974-016-0745-x)
Supplement: Additional file 2: — CSF cytokine concentrations in LNB, non-LNB, TBE, and MS patients. The median concentrations and ranges of each measured cytokine in the CSF of different patient groups are presented. Also the p values of statistical analyses (independent samples t test with Boferroni’s correction) of each studied cytokine are shown. (DOCX 22 kb) [file 12974_2016_745_MOESM2_ESM.docx]

|  | LNB |  | Non-LNB control |  | TBE |  | MS |  | LNB vs Non-LNB control | LNB vs TBE | LNB vs MS | TBE vs Non-LNB control | MS vs Non-LNB control | TBE vs MS |
| --- | --- | --- | --- | --- | --- | --- | --- | --- | --- | --- | --- | --- | --- | --- |
| Cytokine | Median pg/ml | Range | Median pg/ml | Range | Median pg/ml | Range | Median pg/ml | Range | *p*-value | *p*-value | *p*-value | *p*-value | *p*-value | *p*-value |
| IL-1α | 3.4 | 0.1 - 9.7 | 3.8 | 1.5 - 6.2 | 3.0 | 0.8 - 6.4 | 6.3 | 4.0 - 9.4 | 0.272 | 1.00 | <0.001 | 0.1157 | <0.001 | <0.001 |
| IL-1β | 1.4 | 0.3 - 73.9 | 0.3 | 0.2 - 0.8 | 3.6 | 0.0 - 12.9 | 0.3 | 0.2 - 3.2 | <0.001 | 0.2932 | <0.001 | <0.001 | 1.00 | <0.001 |
| IL-1ra | 119.3 | 9.5 - 7443.2 | 2.2 | 2.2 - 9.5 | 281.6 | 14.7 - 1000.7 | 19.2 | 2.8 - 209.1 | <0.001 | 0.1207 | <0.001 | <0.001 | <0.001 | <0.001 |
| IL-2ra | 62.5 | 14.0 - 309.2 | 46.2 | 18.0 - 177.1 | 66.7 | 23.2 - 127.5 | 48.9 | 23.2 - 225.5 | 0.4717 | 1.00 | 1.00 | 0.6798 | 1.00 | 1.00 |
| IL-3 | 237.8 | 105.5 - 1859.3 | 117.2 | 56.0 - 204.0 | 222.6 | 160.9 - 384.3 | 160.9 | 96.4 - 355.6 | <0.001 | 1.00 | <0.001 | <0.001 | 0.0022 | <0.001 |
| IL-4 | 1.2 | 0.2 - 14.8 | 0.2 | 0.2 - 5.6 | 3.0 | 0.6 - 7.1 | 1.3 | 0.1 - 6.1 | <0.001 | <0.001 | 1.00 | <0.001 | <0.001 | 0.0047 |
| IL-6 | 62.4 | 3.9 - 2358.5 | 4.1 | 1.9 - 22.3 | 998.5 | 6.1 - 111250.3 | 4.0 | 1.2 - 11.2 | <0.001 | 0.003 | <0.001 | <0.001 | 1.00 | <0.001 |
| IL-7 | 18.7 | 0.6 - 179.2 | 1.1 | 0.6 - 6.1 | 38.4 | 4.9 - 94.4 | 2.9 | 1.4 - 14.8 | <0.001 | 0.0465 | <0.001 | <0.001 | <0.001 | <0.001 |
| IL-8/  CXCL8 | 465.5 | 134.6 - 16025.4 | 117.1 | 55.2 - 1800.6 | 499.6 | 72.8 - 2676.0 | 107.0 | 38.4 - 210.9 | <0.001 | 1.00 | <0.001 | <0.001 | 1.00 | <0.001 |
| IL-9 | 21.9 | 7.5 - 123.8 | 9.3 | 6.6 - 15.3 | 32.0 | 9.4 - 55.2 | 10.3 | 4.2 - 21.0 | <0.001 | 1.00 | <0.001 | <0.001 | 1.00 | <0.001 |
| IL-10 | 50.6 | 8.4 - 654.4 | 7.8 | 5.7 - 10.3 | 21.6 | 7.6 - 48.0 | 8.4 | 4.2 - 23.2 | <0.001 | <0.001 | <0.001 | <0.001 | 1.00 | <0.001 |
| IL-12(p40) | 790.7 | 419.5 - 2307.6 | 501.1 | 263.8 - 680.2 | 639.0 | 419.5 - 1057.5 | 602.0 | 411.1 - 847.3 | <0.001 | 0.0126 | <0.001 | 0.0033 | 0.0084 | 0.9431 |
| IL-12(p70) | 12.2 | 0.5 - 315.2 | 2.8 | 0.2 - 8.7 | 30.9 | 8.1 - 51.9 | 7.1 | 3.9 - 39.36 | <0.001 | 0.0116 | 0.038 | <0.001 | <0.001 | <0.001 |
| IL-13 | 70.2 | 2.1 - 332.2 | 2.9 | 0.2 - 16.5 | 62.3 | 5.2 - 165.9 | 5.6 | 1.6 - 35.5 | <0.001 | 1.00 | <0.001 | <0.001 | 0.0076 | <0.001 |
| IL-15 | 22.5 | 1.1 - 133.7 | 18.3 | 7.0 - 38.7 | 45.3 | 18.4 - 64.9 | 17.3 | 3.1 - 27.8 | 1.00 | <0.001 | 0.3176 | <0.001 | 1.00 | <0.001 |
| IL-16 | 177.9 | 25.2 - 2096.4 | 31.1 | 2.9 - 59.1 | 143.9 | 29.7 - 474.5 | 29.7 | 1.8 - 202.5 | <0.001 | 0.6141 | <0.001 | <0.001 | 1.00 | <0.001 |
| IL-17 | 90.8 | 6.9 - 560.1 | 6.9 | 6.9 - 296.2 | 135.2 | 41.6 - 244.9 | 74.1 | 3.1 - 273.6 | <0.001 | 0.0062 | 1.00 | <0.001 | <0.001 | 0.0045 |
| IL-18 | 10.9 | 3.2 - 51.3 | 5.2 | 2.2 - 10.0 | 17.2 | 6.5 - 34.5 | 8.2 | 4.8 - 40.1 | <0.001 | 0.123 | 0.017 | <0.001 | <0.001 | <0.001 |
| CXCL1/Groα | 163.3 | 6.1 - 6431.6 | 42.4 | 12.2 - 205.6 | 222.5 | 55.6 - 2448.6 | 55.6 | 38.6 - 130.6 | <0.001 | 1.00 | <0.001 | <0.001 | 0.1927 | <0.001 |
| CXCL9/MIG | 2762.1 | 3.7 - 98942.3 | 76.4 | 16.2 - 1194.8 | 3496.9 | 127.1 - 9431.7 | 121.3 | 25.5 - 554.2 | <0.001 | 1.00 | <0.001 | <0.001 | 0.4663 | <0.001 |
| CXCL10/  IP-10 | 14558.7 | 541.7 - 231061.0 | 468.5 | 93.8 - 2628.4 | 55084.2 | 951.9 - 55084.2 | 1072.8 | 244.9 - 2878.9 | <0.001 | 1.00 | <0.001 | <0.001 | 0.0014 | <0.001 |
| CXCL12α/  SDF-1α | 157.3 | 28.3 - 594.6 | 56.5 | 28.3 - 209.1 | 205.0 | 61.2 - 446.5 | 90.8 | 30.6 - 299.3 | <0.001 | 1.00 | 0.0014 | <0.001 | 0.3683 | <0.001 |
| CCL2/MCP-1/MCAF | 302.6 | 38.6 - 1874.2 | 310.3 | 134.7 - 1287.1 | 297.2 | 66.7 - 1414.7 | 206.3 | 65.1 - 503.1 | 1.00 | 1.00 | 0.0867 | 1.00 | 0.0318 | 1.00 |
| CCL3/  MIP-1α | 5.7 | 0.6 - 55.1 | 0.6 | 0.1 - 1.9 | 7.1 | 2.3 - 10.4 | 1.5 | 0.3 - 6.7 | <0.001 | 1.00 | <0.001 | <0.001 | <0.001 | <0.001 |
| CCL4/  MIP-1β | 65.4 | 21.9 - 376.8 | 28.9 | 17.3 - 79.2 | 51.2 | 19.8 - 81.0 | 35.5 | 17.6 - 61.5 | <0.001 | 0.0381 | <0.001 | <0.001 | 0.1336 | 0.0075 |
| CCL5/RANTES | 64.9 | 0.2 - 1319.6 | 2.0 | 2.0 - 2.0 | 36.3 | 0.2 - 111.0 | 0.2 | 0.2 - 60.7 | <0.001 | 1.00 | <0.001 | <0.001 | 0.0293 | <0.001 |
| CCL11/Eotaxin-1 | 11.6 | 0.1 - 294.7 | 2.7 | 1.4 - 7.0 | 20.2 | 7.9 - 58.3 | 0.1 | 0.1 - 26.0 | <0.001 | 0.0062 | <0.001 | <0.001 | <0.001 | <0.001 |
| CCL27/CTACK | 67.8 | 13.4 - 275.5 | 25.9 | 8.8 - 54.7 | 45.7 | 21.4 - 91.5 | 21.4 | 6.1 - 118.9 | <0.001 | 0.5643 | <0.001 | 0.0249 | 1.00 | <0.001 |
| IFN-α2 | 34.9 | 1.7 - 633.1 | 9.2 | 1.7 - 29.1 | 43.6 | 26.2 - 59.8 | 28.5 | 14.3 - 88.7 | <0.001 | 0.4366 | 1.00 | <0.001 | <0.001 | <0.001 |
| IFN-γ | 28.8 | 1.3 - 1312.8 | 2.7 | 1.3 - 23.9 | 108.8 | 7.7 - 445.8 | 12.8 | 0.5 - 94.6 | <0.001 | <0.001 | <0.001 | <0.001 | <0.001 | <0.001 |
| TNF-α | 21.9 | 0.8 - 774.0 | 0.8 | 0.8 - 8.7 | 59.5 | 7.8 - 186.8 | 7.8 | 1.1 - 67.6 | <0.001 | <0.001 | 0.0024 | <0.001 | <0.001 | <0.001 |
| TNF-β | 3.5 | 0.0 - 25.7 | 2.1 | 0.0 - 4.2 | 1.8 | 0.1 - 10.8 | 2.4 | 0.1 - 20.2 | 1.00 | 1.00 | 1.00 | 1.00 | 1.00 | 1.00 |
| HGF | 256.3 | 0.7 - 735.6 | 120.1 | 53.8 - 281.9 | 372.8 | 175.2 - 938.9 | 232.0 | 89.0 - 683.6 | 0.0819 | 0.0559 | 1.00 | <0.001 | <0.001 | 0.0113 |
| LIF | 24.9 | 10.8 - 94.6 | 20.9 | 12.8 - 30.3 | 26.7 | 14.9 - 37.0 | 20.3 | 12.6 - 59.1 | 0.0613 | 1.00 | 0.185 | 0.0228 | 1.00 | 0.101 |
| M-CSF | 79.5 | 18.5 - 471.6 | 71.5 | 35.9 - 190.9 | 103.3 | 54.2 - 1095.1 | 67.7 | 43.3 - 102.7 | 1.00 | 0.075 | 0.8337 | 0.2872 | 0.8674 | 0.0193 |
| MIF | 818.5 | 19.7 - 4550.8 | 356.7 | 39.4 - 4391.0 | 517.5 | 97.7 - 4740.8 | 223.8 | 51.8 - 789.0 | 0.2905 | 1.00 | <0.001 | 1.00 | 0.1859 | 0.0084 |
| β-NGF | 1.9 | 0.2 - 16.4 | 0.8 | 0.1 - 1.6 | 3.9 | 0.0 - 5.9 | 0.6 | 0.0 - 15.4 | <0.001 | 1.00 | <0.001 | 0.002 | 1.00 | 0.0062 |
| SCF | 87.4 | 5.5 - 348.2 | 51.2 | 24.4 - 91.8 | 99.8 | 44.6 - 136.0 | 41.0 | 22.9 - 114.6 | <0.001 | 1.00 | <0.001 | <0.001 | 1.00 | <0.001 |
| SCGF-β | 18250.6 | 58.8 - 38233.9 | 19774.6 | 6083.9 - 38873.9 | 12153.6 | 4759.9 - 22012.3 | 8672.7 | 1673.8 - 26161.5 | 1.00 | 0.8952 | 0.0158 | 0.0482 | <0.001 | 0.1028 |
| TRAIL | 43.1 | 3.5 - 375.9 | 3.5 | 3.5 - 11.8 | 39.3 | 5.0 - 84.7 | 13.7 | 2.5 - 96.0 | <0.001 | 1.00 | <0.001 | <0.001 | <0.001 | <0.001 |
| PDGF-bb | 26.0 | 2.0 - 161.7 | 6.3 | 0.6 - 22.2 | 51.9 | 11.7 - 89.4 | 6.4 | 1.5 - 34.8 | <0.001 | 0.2297 | <0.001 | <0.001 | 1.00 | <0.001 |
| FGF basic | 54.2 | 3.1 - 248.6 | 13.0 | 3.1 - 57.2 | 93.8 | 37.4 - 131.5 | 35.4 | 14.8 - 75.2 | <0.001 | 0.0108 | 0.0058 | <0.001 | 0.0024 | <0.001 |
| G-CSF | 206.7 | 42.7 - 928.0 | 25.7 | 12.8 - 267.7 | 1747.4 | 64.7 - 5465.6 | 105.7 | 30.2 - 475.9 | <0.001 | <0.001 | 0.0094 | <0.001 | <0.001 | <0.001 |
| GM-CSF | 135.2 | 61.4 - 461.9 | 107.6 | 90.0 - 150.7 | 123.3 | 85.0 - 252.4 | 90.9 | 60.0 - 115.0 | <0.001 | 1.00 | <0.001 | 0.0451 | <0.001 | <0.001 |
| VEGF | 72.7 | 16.1 - 363.7 | 42.5 | 31.5 - 58.4 | 84.0 | 47.0 - 194.9 | 42.5 | 12.6 - 74.5 | <0.001 | 1.00 | <0.001 | <0.001 | 1.00 | <0.001 |
| CXCL13/BCA-1 | 4770.0 | 460.0 - 66108.0 | 3.9 | 3.9 - 22.4 | 15.3 | 3.9 - 406.4 | 3.9 | 3.9 - 280.5 | <0.001 | <0.001 | <0.001 | 0.0033 | 0.0795 | 0.4964 |
